# The Effect of Ageing on Clinical, Hormonal and Sonographic Features Associated with PCOS—A Long-Term Follow-Up Study

**DOI:** 10.3390/jcm10102101

**Published:** 2021-05-13

**Authors:** Małgorzata Jacewicz-Święcka, Sławomir Wołczyński, Irina Kowalska

**Affiliations:** 1 Department of Endocrinology, Diabetology and Internal Medicine, Medical University of Bialystok, 15-089 Białystok, Poland; 2 Department of Reproduction and Gynaecological Endocrinology, Medical University of Bialystok, 15-276 Białystok, Poland; slawek.wolczynski@gmail.com; 3 Department of Internal Medicine and Metabolic Diseases, Medical University of Bialystok, 15-089 Białystok, Poland; irina.kowalska@umb.edu.pl

**Keywords:** PCOS, oligomenorrhea, hyperandrogenism, E-selectin, longitudinal study, miscarriages, fertility, aging

## Abstract

The knowledge of polycystic ovary syndrome (PCOS) natural history is limited. Our objective was to assess the effect of aging on clinical, hormonal and sonographic ovarian PCOS features and additionally to identify parameters that impact the course of PCOS. A secondary aim was to supply additional information on the reproductive outcome in women with previously diagnosed PCOS. A longitudinal cohort study with a median follow-up of 120.9 months was conducted, and 31 Caucasian women previously diagnosed with PCOS according to the Rotterdam criteria were re-examined at a median age of 35. Clinical examinations; transvaginal ultrasound scans; and lipid, E-selectin and sex hormone assessments were performed at the beginning and at the end of the follow-up. It was observed that menstrual cycles became regular and sonographic morphology of ovaries was normalized in 55% and 49% of the participants, respectively (all *p* < 0.05). At the final assessment, 55% of the women no longer met the criteria for PCOS (*p* < 0.05). The age, follicle-stimulating hormone (FSH) and E-selectin assessed at the baseline were the most important predictors of the PCOS persistence into later years (respectively, OR = 0.84, OR = 0.39, OR = 1.08, all *p* < 0.05). Ninety-five percent of the patients who had ever been trying to conceive became pregnant a minimum of once. The women with persistent PCOS had worse metabolic and reproductive parameters compared to the women with resolved PCOS. Positive correlations were found between the number of miscarriages and ovarian volume, LH, androstenedione, 17-hydroxyprogesterone and an increase in E-selectin during the follow-up (*R* = 0.46, *R* = 0.59, *R* = 0.54, *R* = 0.49, *R* = 0.47, all *p* < 0.05). In conclusion, progressing from the third to the fourth decade is connected with a reduction in PCOS features, which seems to have a great impact on fertility of women with a previous diagnosis of PCOS. FSH and E-selectin, as determined at the initial PCOS diagnosis, had an impact on the disappearance of the syndrome years after.

## 1. Introduction

Polycystic ovary syndrome (PCOS) is the most prevalent endocrinopathy affecting reproductive-aged women, with a prevalence of 4–21% depending on the studied population and applied diagnostic criteria [1,2]. Since it was first described by Stein and Leventhal [3], there has been an ongoing discussion related to its definition and etiology [4]. Currently, the criteria reported by ESHRE in Rotterdam in 2003 are the most commonly used in research and clinical care [5]. Rotterdam criteria require the presence of a minimum of two of the following three features to establish a diagnosis of PCOS: clinical or biochemical hyperandrogenism (HA), oligomenorrhea or amenorrhea (OM/AM) and polycystic ovarian morphology (PCOM) by ultrasound, after exclusion of secondary causes. Lately, new evidence-based International Guidelines for the Assessment and Management of PCOS [6] reinforced the 2003 Rotterdam criteria but also revised the cut-off of follicle number per ovary, as equipment advances increased sensitivity of ultrasonography, and provided the possibility to consider androstenedione and dehydroepiandrosterone sulfate (DHEAS) in the diagnostic process if free or total testosterone (TT) are not elevated. Possible combinations of Rotterdam criteria components resulted in the identification of four phenotypes [7]. Published data indicate that the majority of PCOS patients diagnosed within the clinical setting demonstrate phenotype A, that is, all three main PCOS features [2]. The clinical manifestations of PCOS are highly heterogeneous, as PCOS affects multiple aspects of women’s overall health and is associated with reproductive, metabolic and psychological dysfunctions [8].

It is well established that insulin resistance (IR) and compensatory hyperinsulinemia are central etiological abnormalities in women with PCOS. They are responsible for the overproduction of ovarian and adrenal androgens and decreased sex hormone-binding globulin (SHBG) concentration [9,10,11,12,13], which in turn leads to an increase in androgen bioavailability [14,15]. HA represents a main attribute of PCOS [16] as the majority of patients with PCOS exhibit increased levels of androgens or the free androgen index (FAI) calculated as the ratio between TT and SHBG [17,18].

IR and HA lead to unfavorable metabolic profiles in PCOS patients [11,19,20]. Women with PCOS feature higher body mass index (BMI) compared with healthy women, and they have an increased risk of abnormal glucose metabolism, atherogenic dyslipidemia, arterial hypertension, metabolic syndrome and nonalcoholic fatty liver disease compared with BMI-matched non-PCOS women [21,22,23]. IR together with hyperinsulinemia, dyslipidemia and HA plays an important role in inflammatory pathways [24] and has a negative impact on endothelial function [25,26,27]. Injured endothelium secretes numerous adhesion molecules and cytokines responsible for the initiation and progression of the atherosclerotic process [28]. E-selectin is a cell adhesion molecule that regulates adhesive interactions between certain blood cells and endothelium. The role of E-selectin in the pathogenesis of cardiovascular diseases and diabetes has been confirmed [29,30,31]. Elevated concentrations of E-selectin were found in the women with PCOS compared with non-PCOS women [32,33,34], which indicates a subclinical inflammation process in PCOS women.

IR augments luteinizing hormone (LH) [35] resulting in premature luteinization of follicles [36] and disturbance of the LH/follicle-stimulating hormone (FSH) ratio [37]. Obesity, IR, HA and gonadotrophin imbalance probably have an impact on higher anti-Müllerian hormone (AMH) levels in women with PCOS compared to healthy women [35,36,38], which is connected with an increased number of antral follicles and higher production of AMH per antral follicle [39,40]. Measurement of serum AMH was indicated as very useful for identification of PCOS and has even been suggested as a diagnostic criterion [41,42]. Interestingly, it was found that AMH inhibits folliculogenesis [43] and plays an important role in the pathophysiology of oligo-/anovulation associated with PCOS [44,45,46,47]. Decreased rates of ovulation and metabolic disorders both contribute to subfertility in women with PCOS [48]. It was described that PCOS has a nearly 80% prevalence among infertile anovulatory women [49]. However, data concerning reproductive outcome in this group are limited and inconsistent [48,50,51].

The ongoing changes of the diagnostic criteria for PCOS; difficulties associated with diagnosing PCOS in adolescents, as well as in peri- and postmenopause; operator- and equipment-dependent diagnostic examinations; and rapidly advancing progress in the technological and laboratory diagnostic methods make it difficult to address longitudinal studies and to compare data available in the literature. Therefore, very few studies assessing the long-term changes in women with previously confirmed PCOS have been published. Given the limited knowledge on the natural history of PCOS, we conducted a longitudinal follow-up study including 31 women with a previous diagnosis of PCOS according to the Rotterdam criteria. A detailed re-evaluation of participants’ metabolic profiles with particular emphasis on glucose tolerance has been provided recently [52]. The aim of this research was to describe the effects of aging on clinical, hormonal and ovarian characteristics in the studied cohort and to assess the predictors of change in PCOS status. A secondary aim was to supply additional information on the reproductive outcome in women with previously diagnosed PCOS.

## 2. Material and Methods

### 2.1. Study Population

Ninety-one potentially eligible women were identified by the out-patient register at endocrinology and gynecology clinics in Białystok. The diagnosis of PCOS made between 2003 and 2009 and correct results of an oral glucose tolerance test (OGTT) conducted at baseline were the inclusion criteria for the study. Unfortunately, no contact could be found with thirty-four women because of the outdated personal data. Of the remaining women, twenty-six patients were excluded, because they refused to participate in the study (*n* = 19), mainly due to living too far away from Białystok; they were pregnant or within 12 months of delivery (*n* = 3); or they had acute (within the previous 30 days) or chronic infection (*n* = 1), malnutrition (*n* = 1), history of neoplasm or other serious medical problem (*n* = 2). Finally, thirty-one Caucasian women were enrolled in the analysis. There were no statistically significant differences between the studied cohort (*n* = 31) and the women who did not participate in the follow-up examination (*n* = 60) concerning age; BMI; cycle irregularity; FAI; or concentrations of TT, AMH, LH and FSH (all *p* > 0.05). The median age of the participants at the baseline was 25.53 years (interquartile range (IQr) = 21.51–29.22).

### 2.2. Baseline Study

When first examined, the diagnosis of PCOS was made according to the ESHRE 2003 Rotterdam criteria [5]—a minimum of two out of three criteria were fulfilled: PCOM in ultrasound examination, OM/AM, clinical/biochemical HA, after exclusion of related disorders. PCOM was defined as ovarian follicle number (OFN) ≥ 12 (follicles measuring 2–9 mm in diameter) and/or ovarian volume ≥10 mL in at least one ovary. OM was defined as menstrual cycle intervals ≥35 days or <8 cycles per year, while AM was considered when menstrual cycle length was >90 days. Clinical HA was assessed on the basis of the presence of hirsutism (>8 according to modified Ferriman–Gallwey score (mFGS)), acne or androgenic alopecia. Biochemical HA was defined as TT ≥ 0.7 ng/mL and/or FAI ≥ 5. Related disorders like hyperprolactinemia, thyroid dysfunction, ovary neoplasms and nonclassic congenital adrenal hyperplasia were excluded by proper tests.

The recruitment of subjects, anthropometric measurements, clinical examination, an OGTT, sex hormone and lipid assessments and bioelectric impedance analysis were performed as previously described [53]. A euglycemic hyperinsulinemic clamp technique was used to measure insulin sensitivity [54], and the rate of whole-body glucose uptake (M-clamp value) was calculated as the mean glucose infusion rate from 80 to 120 min, corrected for glucose space and normalized per kilogram of fat-free mass [55].

### 2.3. Ethical Approval

All participants provided informed and written consent before conducting the study and after full explanation of the nature and purpose of all procedures used. The study protocol was approved by the Ethics Committee of the Medical University of Białystok and followed the principles of the Declaration of Helsinki (reference no. R-I-002/347/2015; date of approval 24 September 2015).

### 2.4. Follow-Up Study

Thirty-one women were re-evaluated between December 2015 and May 2017. Baseline tests were repeated and PCOS status in each participant was assessed once again.

#### 2.4.1. Protocol of the Study

The comprehensive questionnaire, anthropometric measurements, physical examination, bioelectrical impedance analysis, transvaginal ultrasound scans, blood collection for biochemical tests and an OGTT were all performed for each woman within one day, 3–5 days after spontaneous menstrual bleeding or at random in the presence of AM (after excluding pregnancy using appropriate test). All the participating women declared that for at least three months before an examination they had not received antiandrogens, oral contraceptives (OCs), insulin sensitizers or any other drugs known to affect hormones or carbohydrate metabolism. No woman had a diagnosis of prediabetes or diabetes before being submitted to an OGTT.

#### 2.4.2. Clinical Evaluation

Detailed interviews according to special questionnaire and physical examinations were performed by the same physician for all participants. The questionnaire covered data regarding reproductive history, menstruation, previous and current diseases, past surgeries, smoking, special diets, medication use, obtained education and occupation and family history of diseases (≥1 s degree relative). The presence of hirsutism, androgenic alopecia and acne was verified. Anthropometric and blood pressure measurements, as well as the assessment of fat mass by multi-frequency bioelectrical impedance analysis, were performed as previously described [52].

#### 2.4.3. Image Tests

Transvaginal ultrasound scans of ovaries were performed for thirty patients with a 5–9 MHz transvaginal transducer (Voluson 730 Expert, GE Healthcare, Zipf, Austria). Ovarian volume (OVol) was calculated using the simplified formula for a prolate ellipsoid [56]. OVol, follicle number (OFN) and mean follicle diameter in the right and left ovary were calculated and summarized for both ovaries. Transabdominal ultrasound examination of ovaries was performed for one woman (in this case, only OVol was assessed, without OFN). All the patients were examined by the same experienced gynecologist both at the beginning and at the end of the follow-up.

#### 2.4.4. Biochemical Analyses

Blood samples were drawn between 08:00 and 09:00 a.m., after an overnight fast, and were either analyzed immediately or stored for some tests at −80 °C until analyzed. A standardized 75 g OGTT was performed with the measurements of plasma glucose and serum insulin taken at baseline and 30, 60 and 120 min after glucose ingestion. Fasting blood samples were used to assay total cholesterol (TC), triglycerides (TG), high-density lipoprotein cholesterol (HDL-c), glycated hemoglobin (HbA1c), SHBG, TT, androstenedione (A4), 17-hydroxyprogesterone (17-OHP), dehydroepiandrosterone sulfate (DHEAS), FSH, LH, AMH, estradiol (E2), prolactin, high-sensitivity C-reactive protein (hsCRP) and E-selectin. The methods used in biochemical analyses of glucose, insulin, HbA1c, TC, HDL-c, TG, SHBG, TT, A4, DHEAS and 17-OHP were described previously [52]. Serum FSH, LH and prolactin levels were determined with immunoradiometric method (DIAsource ImmunoAssays S.A., Angleur, Belgium) (MDC for FSH: 0.01 mIU/mL, for LH: 0.2 mIU/mL, for prolactin: 0.35 ng/mL; intra-assay and inter-assay CV for FSH < 2% and 4.4%, for LH < 3.9% and 8%, for prolactin < 5.2% and 9.2%, respectively). AMH concentrations were determined by enzyme immunoassay (Beckmann Coulter, Brea, United States); MDC: 0.08 ng/mL, intra-assay and inter-assay CV < 5.4% and 5.6%. E2 was determined with radioimmunoassay (DIAsource ImmunoAssays, Angleur, Belgium), intra-assay and inter-assay CV < 8% and <13.9%. HsCRP levels were measured by a highly sensitive immunoturbidimetric assay (Cobas c111, Roche Diagnostic Ltd., Basel, Switzerland). E-selectin was detected by enzyme-linked immunosorbent assays using commercially available reagents (R&D Systems Inc., Minneapolis, MN, USA); MDC: 0.027 ng/mL, intra-assay and inter-assay CV < 6.6% and 8.7%, respectively.

#### 2.4.5. Calculations

Homeostasis model assessment score (HOMA), Matsuda index, HOMA β-cell function (HOMA-%β), plasma LDL-c and FAI were calculated as previously described [52].

#### 2.4.6. Statistical Analysis

Lilliefors and Shapiro–Wilk tests were used to analyse all variables for normality of distribution. Values were expressed as median (interquartile range) due to non-normal distribution of data. Nonparametric Mann–Whitney U-test was used to make comparisons between the groups. Comparisons between variables estimated at baseline and after follow-up were evaluated with Wilcoxon signed-rank tests and the sign test. Spearman test was used to perform correlation analysis. Chi-square test was used to analyze differences in categorical variables between groups. Afterward, univariate and multivariate logistic regression analyses were performed. *p*-value < 0.05 was considered statistically significant. Statistica package (Statistica 13.1, Statsoft, Cracow, Poland) and Stata/IC 12.1 (StataCorp LP, College Station, United States) were used to perform the statistical analysis for the study.

#### 2.4.7. Definitions

Diagnosis of PCOS was made according to ESHRE 2003 Rotterdam criteria [5] (description of the criteria available in Section 2.2) to compare data obtained at the beginning and at the end of the follow-up in the studied cohort. ESHRE 2003 Rotterdam criteria were commonly used in the text, unless otherwise stated. Definition of PCOS according to ESHRE 2018 [6] was used to describe the studied group in accordance with the current diagnostic criteria, to compare the differences in the metabolic and hormonal profile between the women with resolved and persistent PCOS at the final examination and to identify predictors of PCOS persistence or disappearance. The diagnostic criteria of PCOS according to ESHRE 2018 are corresponding to ESHRE 2003 criteria, with the only differences in the definition of PCOM (OFN ≥ 20 and/or ovarian volume ≥ 10 mL in at least one ovary) and biochemical HA (TT ≥ 0.7 ng/mL and/or FAI ≥ 5 and/or A4 > 3 ng/mL and/or DHEAS above the level recommended for the respective age group).

Metabolic syndrome was defined according to the International Diabetes Federation (IDF) [57], and a detailed description of the applied criteria was provided previously [52]. Prediabetes referred to impaired fasting glucose (fasting plasma glucose levels: 100–125 mg/dL), impaired glucose tolerance (the 2-h plasma glucose in an OGTT: 140–199 mg/dL) and HbA1c (5.7–6.4%) [58].

## 3. Results

The patients were followed for a median period of 120.9 months (IQr = 107.17–127.38). The median age of the participants at the end of the observation was 35 years (IQr = 31.2–39.8). As previously described, 45% of the participants developed prediabetes and no one developed diabetes during the follow-up [52].

### 3.1. The Progressive Reduction in PCOS Symptoms

The prevalence of OM and PCOM decreased, whereas the prevalence of biochemical and clinical HA did not change significantly during the follow-up observation, and consequently, the percentage of the women who fulfilled PCOS criteria dropped by half (Table 1).

It should be added that if ESHRE 2018 diagnostic criteria for PCOS were used at the final examination, the prevalence of PCOS among the studied cohort would be 58% (*n* = 18) and the prevalence of phenotype A would be 50% (*n* = 9) among the women with persistent PCOS. Moreover, a minimum of one of four studied androgens would be elevated in 77% of the participants (*n* = 24), and 94% of the women (*n* = 29) would feature clinical and/or biochemical HA.

### 3.2. Sex Hormones

Hormonal profile of the participating women, assessed at the beginning of the observation, is shown in Table 2.

It was found that final concentrations of SHBG and AMH, as well as the changes of AMH, E2, LH, FSH and LH/FSH ratio, were significantly correlated with baseline values of these parameters (respectively, *R* = 0.57, *p* = 0.0008; *R* = 0.60, *p* = 0.0004; *R* = −0.57, *p* = 0.0008; *R* = −0.54, *p* = 0.002; *R* = −0.69, *p* = 0.00002; *R* = −0.55, *p* = 0.001; *R* = −0.53, *p* = 0.002). Additionally, ∆AMH, final 17-OHP and final LH/FSH ratio were negatively correlated with age (respectively, *R* = −0.48, *p* = 0.006; *R* = −0.46, *p* = 0.01; *R* = −0.47, *p* = 0.007).

The normalization of TT and FAI levels was found respectively in 77% and 53% of the women who had elevated values of these parameters at the baseline. De novo elevated levels of TT and FAI were observed in almost 10% and 13% of the studied women, respectively. Thus, the percentage of the women with biochemical HA (defined as elevated TT or FAI) decreased from 61% to 42% in the entire cohort. Interestingly, at the final examination TT ≥ 0.7 ng/mL was found only in the women younger than 35 years old, and the percentage of the young women with elevated TT totaled 35% (*p* = 0.01). Moreover, elevated TT was found in 36% of the women with BMI < 25 kg/m^2^ and in 6% of the women with higher BMI (*p* = 0.04). Detailed correlations between HA (with particular focus on FAI) and metabolic parameters were described previously [52]. It is worth emphasizing that all the women with TT ≥ 0.7 ng/mL and 36% of the women with lower TT disclosed PCOM in ultrasonography (*p* = 0.005) and that 69% of the women with elevated TT or FAI compared to 31% of the women without biochemical HA exhibited OM (*p* = 0.009).

A4, DHEAS and 17-OHP were assessed in all the participants only at the end of the follow-up, and their median values totalled 4.26 ng/mL (IQr: 2.92–5.27), 223.1 µg/dL (IQr: 166.2–299.3) and 1.24 ng/mL (IQr: 0.97–1.59), respectively. A4 and DHEAS were elevated in 71% and 10% of the participants, respectively, and they were both correlated with final AMH (respectively, *R* = 0.36, *R* = 0.42, all *p* < 0.05). Moreover, A4 and 17-OHP were observed to be correlated with final LH/FSH ratio (respectively, *R* = 0.65, *R* = 0.58, all *p* < 0.001).

### 3.3. Clinical HA

The percentage of the patients with clinical features of HA decreased slightly from 52% (*n* = 16) to 42% (*n* = 13); however, 62% of the women who had clinical HA in the past still had it after the follow-up, and 20% of the women who did not have clinical HA at the baseline developed it during the observation (*p* = 0.02). As expected, the women with clinical HA had more points in mFGS compared to the women without clinical HA (respectively, 10 vs. 4, *p* = 0.0001). Moreover, the participants with clinical HA were more obese and exhibited higher percentage of HbA1c at the end of the follow-up in comparison with the women without clinical HA (respectively, BMI: 33.24 vs. 23.65 kg/m^2^, *p* = 0.02; HbA1c: 5.4% vs. 5.15%, *p* = 0.01).

### 3.4. OM

During the follow-up, a decrease was noted in the percentage of the patients with OM from 97% (*n* = 30) to 42% (*n* = 13) (*p* = 0.0001). At the final examination, OM was reported by 59% of the women younger than 35 years old and by 21% of older women (*p* = 0.04). The normalization of the menstrual cycles occurred more often in the women who at the first evaluation had featured BMI < 25 kg/m^2^ (79% vs. 41%, *p* = 0.04), M-clamp value higher than estimated median (81% vs. 33%, *p* = 0.007), HOMA-IR < 2.5 (79% vs. 41%, *p* = 0.04) and FAI < 5 (75% vs. 40%, *p* = 0.04). Furthermore, the women with normalized menstrual cycles exhibited significantly lower AMH, LH/FSH, hsCRP and E-selectin both at the beginning and at the end of the observation and lower final TG, OFN and systolic blood pressure values compared to the participants whose menstrual cycles remained irregular (all *p* < 0.05). Although TT did not differ among the groups at the first evaluation, at the second one it was significantly higher in the women with sustained OM. At the end of the follow-up, increased TT or FAI was found in 69% of the women with OM and in 22% of the regularly menstruating women (*p* = 0.009). PCOM was twice more prevalent among the women with OM than those with regular menses (69% vs. 33%, *p* < 0.05). A detailed comparison between the women with normalized menstrual pattern and those with sustained OM is displayed in Table 3.

### 3.5. PCOM

At the baseline, PCOM was identified in 97% of the participants (*n* = 30), whereas at the end of the follow-up it was observed in 48% of the women (*n* = 15) (*p* = 0.0003). The main hormonal and clinical differences between the women whose morphology of ovaries normalized during the follow-up and the women with persistent PCOM are shown in Table 4. As it is presented, the women whose morphology of ovaries in ultrasonography normalized over time were significantly older and had lower final concentrations of androgens, AMH and E-selectin in comparison with the participants with sustained PCOM. Moreover, the women with normalized morphology of ovaries had higher FSH compared to the participants with PCOM both at the baseline and at the end of the follow-up. Interestingly, no significant differences were found among the women with and without PCOM in the anthropometric and metabolic parameters.

At the end of the follow-up, median values of OFN and OVol in the studied cohort totaled 16.5 (IQr = 11–23) and 10.64 mL (IQr = 7.30–14.56), respectively. Unfortunately, only the presence or lack of PCOM (without detailed data concerning OFN or OVol) had been noted at the first examination.

Significant correlations were found between OFN and final AMH, A4, 17-OHP and E-selectin (respectively, *R* = 0.76, *p* < 0.00001; *R* = 0.43, *p* = 0.02; *R* = 0.41, *p* = 0.02, *R* = 0.52, *p* = 0.004). OVol was correlated with final E-selectin and baseline FSH (respectively, *R* = 0.54, *R* = −0.50, all *p* < 0.01). Moreover, significant correlation was noted between final E-selectin and both final AMH and baseline FSH (respectively, *R* = 0.47; *R* = −0.54, all *p* < 0.01).

### 3.6. Persistent PCOS and Resolved PCOS

The percentage of the women who fulfilled criteria for PCOS in the studied cohort decreased from 100% (*n* = 31) to 45% (*n* = 14) at the end of the follow-up (*p* = 0.0001). Phenotype A was the most commonly found PCOS phenotype in the studied cohort both at the baseline and at the final examination (Table 1).

The women with persistent PCOS were characterized by lower FSH; higher LH/FSH ratio, AMH and TG; and worse values of IR indices both at the baseline and at the end of the observation compared to the participants with resolved PCOS (*p* < 0.05). It was found that at the beginning of the observation, obesity had been more prevalent in the women who at the end of the follow-up were still diagnosed with PCOS compared to the women with resolved PCOS (50% vs. 12%, *p* = 0.02). The comparison of selected parameters between the women with persistent and resolved PCOS is depicted in Table 5.

It is worth mentioning that slightly different results were obtained when PCOS was defined according to ESHRE 2018 criteria. There was a significant difference in age—women with persistent PCOS were significantly younger compared to women with resolved PCOS (34.2 vs. 38.1, *p* = 0.01). Furthermore, differences in the metabolic parameters between the studied groups partly disappeared—only final values of HOMA-%β and systolic blood pressure were significantly higher in the women with persistent PCOS compared to the women with resolved PCOS (all *p* < 0.05). Reproductive hormones and ovarian parameters varied in a similar way between the groups. Additionally, significantly higher values of A4 and DHEAS were found in the women with persistent PCOS compared to the women with resolved PCOS. Interestingly, a significant difference was found between the studied groups in concentrations of E-selectin: higher concentrations of E-selectin were observed both at the baseline and at the end of the follow-up in the women who at the end of the observation no longer met the criteria for PCOS.

Among 31 women with previously diagnosed PCOS, the connections between metabolic, hormonal and inflammation status noted at the beginning of the observation and the presence of PCOS diagnosis according to ESHRE 2018 criteria after a 10-year follow-up period were assessed. The results show that the age and serum concentrations of FSH, E-selectin and AMH determined at the initial PCOS diagnosis are the most important predictors of PCOS persistence in the fourth decade of life (Table 6).

The results of a multivariate logistic regression analysis show that a 1-year increase in the age of the women with PCOS is associated with a nearly 2-fold lower risk of the persistence of PCOS 10 years after initial diagnosis, adjusted for baseline concentrations of FSH and AMH (OR = 0.59; 95% CI: 0.36 to 0.95, *p* = 0.03). Moreover, a one-unit increase in FSH concentration determined in young women at the initial PCOS diagnosis is associated with an over 5-fold lower risk of the persistence of PCOS after 10 years, adjusted for the age and baseline AMH (OR = 0.19; 95% CI: 0.05 to 0.75, *p* = 0.02). Finally, a one-unit increase in the concentration of AMH in the young women at the initial PCOS diagnosis is connected with a 37% higher risk of the persistence of PCOS in their thirties, adjusted for the age and baseline FSH (OR = 1.37; 95% CI: 0.98 to 1.90, *p* = 0.06).

### 3.7. Reproductive Health

#### 3.7.1. Oral Contraceptives

Eighty-one percent of the studied women (*n* = 25) had a history of OC taking. Nineteen percent of the participants (*n* = 6) were on OC treatment while they were contacted at the follow-up, but all of them declared drug withdrawal lasting a minimum of 3 months before the final examination. The variable duration of therapy and different composition of drugs during different periods excluded the possibility of a comprehensive evaluation of the effects of treatment on the evolution of PCOS with age.

#### 3.7.2. Pregnancy

Sixty-eight percent of the studied women (*n* = 21) became pregnant minimum once, which is 95% of the patients who had ever been trying to conceive (*n* = 22). The median age of the studied women at the first pregnancy was 29. The median period of attempts to conceive in the entire group lasted 12 months (IQr: 2–48). It was noted that hsCRP measured at the beginning of the observation and LH/FSH ratio assessed at the final examination were significantly correlated with the number of months of attempts to conceive (respectively, *R* = 0.50, *p* = 0.03; *R* = 0.52, *p* = 0.02). Additionally, the period to conceive was significantly longer in the women with sustained OM compared to the women whose menstrual cycle normalized during the follow-up (respectively, 42 months (IQr: 18–66) vs. 2 months (IQr: 1–12), *p* = 0.02). Seven women underwent assisted reproduction (ART) which was successful in six of them. Considering only women who were trying to conceive, it was noted that 14% of the women with resolved PCOS and 62% of the women with persistent PCOS underwent ART treatment (*p* = 0.02). Only one woman reported complications in pregnancy (gestational diabetes mellitus).

#### 3.7.3. Delivery

Nineteen women gave birth to 26 live healthy children, whose median body mass was 3450 g. No one had more than two children. The median length of the first gestation was 39 weeks. The number of Caesarean sections was 2-fold higher than natural deliveries (17 vs. 9). The number of deliveries was higher in the women with normal morphology of ovaries compared to the women with PCOM (1 (IQr: 1–2) vs. 0 (IQr: 0–1), *p* = 0.02).

#### 3.7.4. Miscarriage

Thirty-three percent of the women who had ever been pregnant reported a minimum of one pregnancy loss. It was observed that the number of miscarriages was positively correlated with OVol, 17-OHP, A4, LH and LH/FSH ratio, all assessed at the final examination (respectively, *R* = 0.46, *p* = 0.04; *R* = 0.49, *p* = 0.03; *R* = 0.54, *p* = 0.01; *R* = 0.59, *p* = 0.005; *R* = 0.56, *p* = 0.008), and with an increase in E-selectin during the follow-up (*R* = 0.47, *p* = 0.03). Furthermore, significant correlations were noted between the number of miscarriages and metabolic parameters assessed at the beginning of the follow-up, such as BMI, waist circumference, fat mass (%) or M-clamp value (respectively, *R* = −0.66, *p* = 0.001; *R* = −0.61, *p* = 0.003; *R* = −0.72, *p* = 0.0002; *R* = 0.63, *p* = 0.002).

## 4. Discussion

The main finding of the present study was that aging in women with PCOS is associated with the improvement of major PCOS features. A decrease by half in the prevalence of PCOM and an increase in the percentage of women with regular menstrual cycles were demonstrated. Fifty-five percent of the studied women did not fulfill the criteria for PCOS anymore at the end of the observation.

Female aging involves a reduction in the pool of growing antral follicles [59,60], which has various consequences for healthy women and women with PCOS [61,62,63,64] due to primary hormonal imbalance in PCOS. Progressive reduction in antral follicle number results in the diminishment of typical ultrasound features of PCOS [65,66] and in a decrease in inhibin B and AMH levels [67,68]. After a fall in inhibin B, FSH increases [69], which enables the full follicle maturation and shortens menstrual cycles [70,71,72]; hence, the incidence of regular menstrual cycles among women with PCOS increases with increasing age [61,62,73]. Furthermore, it seems that aging results in the decline in serum androgen levels in PCOS women [61,74,75,76,77,78]; however, it has been described so far only in a few longitudinal studies [64,79,80]. Extenuation of HA is probably the result of simultaneous diminishment in the pool of growing antral follicles, decrease in adrenal gland production and exhaustion of pancreatic cells [52,74,81].

Amelioration of the PCOS phenotype with aging, in the form of menstrual cycle normalization and a decrease in androgen concentrations, has a great impact on fertility of the women with a previous diagnosis of PCOS [82]. Additionally, a number of reports revealed an increased ovarian reserve and higher AMH concentrations in young women with PCOS compared to age-matched controls [67,83,84]. It was showed that despite serum AMH decreases over time in all the women, a decrease in the PCOS patients is less pronounced and may suggest better preserved ovarian reserve and hence a sustained reproductive life span [63]. Moreover, some data confirm that the anovulatory women with PCOS may become ovulatory with aging [62,85]. We report a high delivery rate in women with a previous diagnosis of PCOS (86%), which is concordant with other studies [51,86,87,88]. Data concerning fertility of Polish women of childbearing age revealed that the mean number of children is 1.51 [89].

Detailed assessment showed that the period of attempts to conceive was longer and ART was more often used in the women with persistent PCOS compared to the participants with resolved PCOS. Furthermore, positive correlations were found between the number of miscarriages and OVol, 17-OHP, A4 and LH, suggesting that the women with PCOM and elevated adrenal androgens can be at a higher risk of miscarriages compared to the women with a previous diagnosis of PCOS but no features of PCOM or adrenal HA. Unless it has been proved that PCOS may independently from BMI increase the risk of miscarriage [50,90,91], the direct causes of this implication are poorly recognized. Several potential risk factors of miscarriages in PCOS have been described. In 1988, Sagle et al. [92] reported a high prevalence of PCOM in women who had recurrent miscarriages, which was later confirmed by other authors [93]. Additionally, available data emphasize the impact of HA on miscarriages [94,95,96,97], but the role of adrenal androgens has not been studied widely [98,99,100]. Moreover, reports concerning the predictive value of LH on miscarriage are distinct [93,101,102]. Recent studies have highlighted thrombophilic disorders as being a potential cause of recurrent miscarriages in women with PCOS [103,104]. To sum up, the connections between clinical and biochemical PCOS features and increased risk of miscarriages need further research.

So far, only a few longitudinal studies evaluating changes in PCOS features have been published, and therefore, the natural history of PCOS is unclear. To our best knowledge, this is the first longitudinal study in which the biochemical predictors of PCOS persistence were assessed. We found that serum FSH, E-selectin and AMH concentrations determined in the young women at the first diagnosis of PCOS may announce or impact the further course of the syndrome. Moreover, we are the first to describe connections between E-selectin and ovarian morphology, sex hormones and the number of miscarriages. Although low-grade chronic inflammation with increased levels of E-selectin in PCOS women compared to controls have been reported already [105,106], the role of E-selectin in the PCOS phenotype, the course of PCOS and fertility has not been described so far. Further research is needed to explain this phenomenon. Interestingly, we found no metabolic parameter to affect the changes in PCOS profile, which may confirm that metabolic complications in PCOS are secondary to hormonal imbalance.

The main limitation of the present study is the small cohort size which precludes extensive analysis of aging in the women with PCOS according to the specific phenotypes. A consecutive shortcoming is the fact that the sample was recruited from an outpatient clinic setting which may contain women with more severe disease compared to the entire community. Additionally, we do not possess information about dietetic habits among the participants and detailed data concerning OFN and OVol that had been determined at the baseline examination. Strengths of our study include the longitudinal design, appropriately diagnosed PCOS in the studied cohort, clearly defined inclusion and exclusion criteria and detailed biochemical assessment. Moreover, the endocrine status in participants was comparable as all the women ceased taking OC and metformin prior to measurements and all the tests were performed on the same day in each woman, 3–5 days after menstrual bleeding or at random in the presence of amenorrhea. Another strength of this research is the fact that evaluation of ovary images in the ultrasonographic examination was accomplished by the same practiced gynecologist for all the participants both at the baseline and at the end of the follow-up.

## 5. Conclusions

In conclusion, there is a paucity of studies with longitudinal follow-up in which detailed assessment of clinical data, hormonal profile and ovarian morphology in women with a previous diagnosis of PCOS was done. Our study demonstrates that the PCOS reproductive phenotype is ameliorated with aging. Moreover, determinations of serum E-selectin, AMH and FSH concentrations in the young women with PCOS can be used to predict the possibility of the PCOS features withdrawal years after initial diagnosis. The role of E-selectin in the PCOS phenotype requires further research. It appears that women with persistent PCOS are at a higher risk of subfertility and adverse metabolic profile compared to women with resolved PCOS.

## Figures and Tables

**Table 1:** Prevalence of major PCOS features at the beginning and at the end of the follow-up (*n* = 31).

Characteristic	Baseline	Follow-Up
PCOS *	100% (31)	45.16% (14) **^a^**
Phenotype A **	70.97% (22)	57.14% *** (8)
Hyperandrogenism	77.42% (24)	67.74% (21)
Biochemical hyperandrogenism	61.29% (19)	41.93% (13)
Increased TT	41.94% (13)	19.35% (6)
Increased FAI	48.39% (15)	35.48% (11)
Clinical hyperandrogenism	51.61% (16)	41.93% (13)
Hirsutism	45.16% (14)	32.26% (10)
Acne	48.39% (15)	16.13% (5) **^a^**
Alopecia	3.23% (1)	3.23% (1)
Oligomenorrhea	96.77% (30)	41.93% (13) **^a^**
Polycystic ovarian morphology	96.77% (30)	48.39% (15) ^a^

Data are expressed as percentage (numbers). Statistical significance was assessed for the comparison of baseline and follow-up parameters. **^a^** Statistically significant (*p* < 0.05). * PCOS defined according to ESHRE 2003. ** Phenotype A defined as coexistence of OM/AM, HA and PCOM. *** Percentage of women with persistent PCOS. Abbreviations: FAI, free androgen index; PCOS, polycystic ovary syndrome; TT, total testosterone.

**Table 2:** Hormonal profile at the beginning of the follow-up (*n* = 31).

Characteristic	Concentration/Value
LH (mIU/mL)	7.8 (4.9–10.8)
FSH (mIU/mL)	5.9 (4.7–7.19)
LH/FSH	1.3 (0.94–1.96)
TT (ng/mL)	0.63 (0.46–0.88)
SHBG (nmol/l)	42.6 (23.68–61.5)
FAI	4.63 (3.67–10.3)
Estradiol (pg/mL)	49.0 (34.0–66.0)
Prolactin (ng/mL)	12.43 (9.0–18.5)
AMH (ng/mL)	9.24 (6.35–12.62)

Data are expressed as median (25–75% quartiles). Abbreviations: AMH, anti-Müllerian hormone; FAI, free androgen index; FSH, follicle-stimulating hormone; LH, luteinizing hormone; SHBG, sex hormone-binding globulin; TT, total testosterone.

**Table 3:** Characteristics of the women according to menstrual pattern assessed at the end of the follow-up.

Characteristic	Regular Menses *de novo* (*n* = 17)	Sustained OM (*n* = 13)	*p*-Value
Age at follow-up (years)	37.60 (35.0–42.2)	34.4 (29.7–34.9)	0.07
BMI at follow-up (kg/m^2^)	24.8 (21.07–31.69)	32.47 (23.98–39.13)	0.04
BMI at baseline (kg/m^2^)	22.85 (21.45–27.82)	29.14 (25.46–36.77)	0.07
Waist circumference at follow-up (cm)	84 (75–97)	105 (90–126)	0.01
Waist circumference at baseline (cm)	77 (71–80)	93 (79–110)	0.02
Fat mass at follow-up (%)	31.3 (22.7–37.5)	45 (32.7–50)	0.008
Fat mass at baseline (%)	32 (27.5–38.5)	42 (29.6–48.1)	NS
Glucose 0′ at follow-up (mg/dL)	90 (89–102)	101 (94.00–103.00)	NS
Glucose 0′ at baseline (mg/dL)	83 (78–89)	86.1 (81–88)	NS
Mean glucose at follow-up (mg/dL)	102.25 (97.25–123.75)	138.5 (123–152)	0.004
Mean glucose at baseline (mg/dL)	100.5 (91.25–113)	118 (102.25–125.75)	NS
Insulin 0′ at follow-up (uIU/mL)	7.73 (6.15–11.54)	16.75 (9.44–19.57)	0.008
Insulin 0′ at baseline (uIU/mL)	10.53 (7.4–14.54)	18.5 (11.7–25.8)	0.05
Mean insulin at follow-up (uIU/mL)	45.61 (35.55–52.66)	78.09 (58.64–105.37)	0.02
Mean insulin at baseline (uIU/mL)	54.5 (37.55–83.25)	102.15 (66.63–116.93)	0.02
M-clamp value at baseline (mg/kgffm/min)	9.84 (8.4–11.99)	5.83 (4.17–7.92)	0.006
Matsuda index at follow-up	5.27 (3.06–6.56)	1.99 (1.57–3.41)	0.008
Matsuda index at baseline	4.23 (2.71–6.42)	1.83 (1.59–3.4)	0.02
Prediabetes at follow-up n (%)	5 (29.41%)	9 (69.23%)	0.03
Triglycerides at follow-up (mg/dL)	63 (50–97)	113 (74–210)	0.01
Triglycerides at baseline (mg/dL)	74 (51–92)	109.2 (76–172)	NS
LDL-c at follow-up (mg/dL)	111.4 (100.4–117.6)	104 (101–110)	NS
LDL-c at baseline (mg/dL)	92.8 (78.2–139.8)	99 (88.4–127.4)	NS
HDL-c at follow-up (mg/dL)	68.0 (56.0–71.0)	50 (42–100)	NS
HDL-c at baseline (mg/dL)	58.2 (53.0–69.0)	56 (43–66)	NS
HsCRP at follow-up (mg/L)	0.38 (0.26–1.06)	3.49 (1.09–6.16)	0.003
HsCRP at baseline (mg/L)	0.94 (0.14–1.61)	2.13 (1.47–4.76)	0.01
E-selectin at follow-up (ng/mL)	10.6 (9.3–12.49)	16.42 (13.22–30.67)	0.009
E-selectin at baseline (ng/mL)	27.2 (16.72–36.15)	39.23 (26.59–55.94)	0.04
Systolic blood pressure at follow-up (mmHg)	122 (109–125)	130 (122–135)	0.01
Systolic blood pressure at baseline (mmHg)	120 (110–125)	120 (110–130)	NS
Metabolic syndrome according to IDF at follow-up n (%)	3 (17.65%)	8 (61.54%)	0.01
Metabolic syndrome according to IDF at baseline n (%)	1 (5.88%)	5 (38.46%)	0.03
FSH at follow-up (mIU/mL)	6.65 (5.5–7.99)	5.08 (4.55–5.26)	0.02
FSH at baseline (mIU/mL)	6.9 (5.6–7.6)	4.9 (3.64–5.9)	0.04
LH/FSH at follow-up	0.65 (0.49–0.87)	1.27 (0.81–1.88)	0.004
LH/FSH at baseline	1.14 (0.79–1.44)	1.96 (1.29–2.26)	0.01
TT at follow-up (ng/mL)	0.39 (0.32–0.48)	0.58 (0.5–0.74)	0.01
TT at baseline (ng/mL)	0.57 (0.45–0.71)	0.77 (0.61–1.06)	NS
SHBG at follow-up (nmol/L)	51.79 (37.66–66.32)	29.34 (23.36–105.09)	NS
SHBG at baseline (nmol/L)	45.13 (34.35–60.90)	27.20 (20.45–54.42)	NS
AMH at follow-up (ng/mL)	2.51 (1.49–3.45)	8.14 (7.44–12.13)	0.0009
AMH at baseline (ng/mL)	7.24 (5.46–9.45)	11.28 (9.91–13.88)	0.04
Attempts of pregnancy ^a^ (months)	2 (1–12) (*n* = 11)	42 (18–66) (*n* = 8)	0.02

OM, oligomenorrhea; SHBG, sex hormone-binding globulin; TT, total testosterone. Data are expressed as median (25–75% quartiles) or numbers (%). *p* < 0.05 was considered statistically significant. ^a^ The period between the beginning of trying for a baby and confirmation of a pregnancy, assessed only in the women who gave birth. Abbreviations: AMH, anti-Müllerian hormone; BMI, body mass index; FSH, follicle-stimulating hormone; HDL-c, high-density lipoprotein cholesterol; HOMA-%β, homeostasis model assessment β-cell function; hsCRP, high-sensitivity C-reactive protein; IDF, International Diabetes Federation; LDL-C, low-density lipoprotein cholesterol; LH, luteinizing hormone; M-clamp value, insulin sensitivity estimated with the euglycemic hyperinsulinemic clamp technique; NS, statistically nonsignificant.

**Table 4:** Characteristics of the women according to ovarian morphology assessed at the end of the follow-up.

Characteristic	Normalized Ovarian Morphology (*n* = 15)	Persistent PCOM (*n* = 15)	*p*-Value
Age at follow-up (years)	38.1 (35–42.2)	33.6 (28–34.9)	0.004
Age of first menorrhea (years)	13 (12–15)	12 (12–13)	0.07
FSH at follow-up (mIU/mL)	6.64 (5.26–8.2)	4.92 (4.54–5.88)	0.04
FSH at baseline (mIU/mL)	6.90 (5.6–8.27)	5 (3.64–5.9)	0.006
LH/FSH at follow-up	0.70 (0.49–1.07)	0.84 (0.75–1.8)	0.07
LH/FSH at baseline	1.14 (0.83–1.46)	1.66 (1.1–2.46)	0.04
TT at follow-up (ng/mL)	0.39 (0.29–0.53)	0.58 (0.44–0.89)	0.01
∆TT (ng/mL)	−0.34 (−0.72–(−0.06))	−0.01 (−0.18–0.22)	0.003
FAI at follow-up	2.25 (1.20–2.80)	6.83 (2.16–10.01)	0.04
∆FAI	−2.06 (−6.68–(−0.80))	−0.5 (−1.57–2.3)	0.05
A4 at follow-up (ng/mL)	3.75 (2.65–4.57)	4.92 (3.84–5.68)	0.02
OHP-17 at follow-up (ng/mL)	1.03 (0.89–1.38)	1.4 (1.16–1.72)	0.04
E2 at follow-up (pg/mL)	50.75 (35.5–84.44)	73.82 (63.21–91.28)	0.06
∆E2 (pg/mL)	9.27 (–0.20–38.32)	34.82 (−4.08–48.57)	NS
AMH at follow-up (ng/mL)	2.20 (1.03–2.94)	8.57 (5.46–14.1)	0.00002
∆AMH (ng/mL)	−5.26 (−7.04–(−2.31))	−2.03 (−4.88–0.79)	0.06
Ovarian follicle number at follow-up (*n* = 30)	11 (9–14)	23.5 (19–28)	0.00001
Ovarian volume at follow-up (mL)	7.49 (5.87–9.6)	13.7 (12.39–21.09)	0.0004
Oligomenorrhea at follow-up n (%)	3 (20%)	9 (60%)	0.03
↑TT/FAI at follow-up n (%)	3 (20%)	10 (66.67%)	0.01
E-selectin at follow-up (ng/mL)	10.60 (6.18–12.3)	16.65 (12.18–18.69)	0.002
E-selectin at baseline (ng/mL)	25.76 (15.27–37.15)	35.02 (26.59–46.20)	0.06

Data are expressed as median (25–75% quartiles) or numbers (%). *p* < 0.05 was considered statistically significant. Abbreviations: A4, androstenedione; AMH, anti-Müllerian hormone; FAI, free androgen index; FSH, follicle-stimulating hormone; LH, luteinizing hormone; NS, statistically nonsignificant; PCOM, polycystic ovarian morphology in ultrasonography; TT, total testosterone; 17-OHP, 17-hydroxyprogesterone.

**Table 5:** Characteristics of the women according to PCOS * status assessed at the end of the follow-up.

Characteristic	Resolved PCOS (*n* = 17)	Persistent PCOS (*n* = 14)	*p*-Value
Age at follow-up (years)	37.6 (35–42.2)	34.2 (30.2–34.9)	0.05
BMI at follow-up (kg/m^2^)	25.56 (21.07–28.83)	33.38 (21.98–39.13)	0.08
BMI at baseline (kg/m^2^)	23.80 (21.45–27.82)	30.09 (21.77–36.77)	NS
Waist circumference at follow-up (cm)	86 (76–95)	107.5 (80–126)	0.04
Waist circumference at baseline (cm)	78 (71–85)	95 (71–110)	NS
Fat mass at follow-up (%)	31.3 (22.7–37.5)	41.5 (27.7–50)	0.03
Fat mass at baseline (%)	34.0 (27.5–38.5)	38.05 (27.5–48.1)	NS
Glucose 0′ at follow-up (mg/dL)	91 (89–102)	96 (90–103)	NS
Glucose 0′ at baseline (mg/dL)	81 (78–86)	86.55 (83–94.4)	0.08
Mean glucose at follow-up (mg/dL)	104.75 (97.25–147.25)	125.63 (109.5–147)	NS
Mean glucose at baseline (mg/dL)	101.5 (91.25–114.75)	112.39 (96–123.25)	NS
Insulin 0′ at follow-up (uIU/mL)	8.2 (6.15–11.54)	16.54 (7.3–20.31)	0.02
Insulin 0′ at baseline (uIU/mL)	10.53 (7.4–14.54)	16.22 (11.7–25.8)	0.04
Mean insulin at follow-up (uIU/mL)	45.8 (35.62–52.66)	69.25 (35.68–105.37)	0.08
Mean insulin at baseline (uIU/mL)	54.5 (37.55–83.25)	84.81 (63.26–116.93)	0.09
Matsuda index at follow-up	5.2 (3.06–6.56)	2.07 (1.7–5.62)	0.07
Matsuda index at baseline	4.23 (2.71–6.42)	2.29 (1.8–4.02)	0.04
HOMA-IR score at follow-up	1.82 (1.35–2.71)	4.15 (1.52–5.09)	0.03
HOMA-IR score at baseline	2.24 (1.41–2.99)	3.52 (2.51–5.91)	0.02
HOMA-%β at follow-up	90.31 (75.2–109.31)	141.57 (115.16–192.38)	0.006
HOMA-%β at baseline	190.29 (164.77–346.5)	252.44 (195.88–298.59)	NS
Prediabetes at follow-up n (%)	7 (41%)	7 (50%)	NS
Triglycerides at follow-up (mg/dL)	63 (50–83)	116 (58–210)	0.03
Triglycerides at baseline (mg/dL)	65 (48.6–86)	120.1 (76–172)	0.02
HDL-c at follow-up (mg/dL)	69 (56–73)	52.5 (42–85)	NS
HDL-c at baseline (mg/dL)	63 (53–69)	56 (43–66.2)	NS
Systolic blood pressure at follow-up (mmHg)	122 (110–125)	127 (116–132)	NS
Systolic blood pressure at baseline (mmHg)	120 (110–125)	120 (110–130)	NS
LH at follow-up (mIU/mL)	4.53 (3.78–5.56)	4.68 (3.81–9.91)	NS
LH at baseline (mIU/mL)	7.7 (4.5–10.62)	8.3 (5.5–11.2)	NS
FSH at follow-up (mIU/mL)	6.64 (5.5–7.99)	4.86 (4.54–5.26)	0.01
FSH at baseline (mIU/mL)	6.9 (5.6–8.01)	4.95 (3.64–5.9)	0.01
LH/FSH at follow-up	0.7 (0.49–0.87)	1.05 (0.76–1.88)	0.02
LH/FSH at baseline	1.1 (0.83–1.44)	1.81 (1.29–2.26)	0.01
TT at follow-up (ng/mL)	0.37 (0.32–0.48)	0.63 (0.5–0.89)	0.0002
TT at baseline (ng/mL)	0.63 (0.46–0.73)	0.63 (0.52–0.88)	NS
SHBG at follow-up (nmol/L)	56.75 (43.35–70.97)	32.44 (23.36–87.35)	0.09
SHBG at baseline (nmol/L)	45.13 (31.62–60.90)	31.98 (20.45–61.5)	NS
FAI at follow-up	2.2 (1.36–2.44)	8.12 (3.53–10.01)	0.006
FAI at baseline	4.3 (3.74–7.74)	5.16 (3.67–10.34)	NS
A4 at follow-up (ng/mL)	3.75 (2.65–4.69)	5.06 (3.84–6.32)	0.01
DHEAS at follow-up (µg/dL)	179.3 (155–281)	253.15 (217.6–299.3)	NS
17-OHP at follow-up (ng/mL)	1.03 (0.89–1.38)	1.32 (1.1–1.72)	0.07
AMH at follow-up (ng/mL)	2.51 (1.49–3.45)	8.68 (6.36–14.1)	0.0003
AMH at baseline (ng/mL)	7.24 (5.46–9.45)	11.39 (8.39–14.1)	0.02
E-selectin at follow-up (ng/mL)	11.34 (10.49–16.80)	14.4 (12.14–18.69)	NS
E-selectin at baseline (ng/mL)	27.20 (16.72–37.15)	30.91 (26.59–46.2)	NS
Ovarian follicle number at follow-up (*n* = 30)	12 (10–17)	23 (17–28)	0.003
Ovarian volume at follow-up (mL)	8.3 (7.24–12.8)	13.4 (10.13–20.4)	0.04
Attempts of pregnancy ^a^ (months)	2.5 (1.25–12) *n* = 12	48 (24–84) *n* = 7	0.02

Data are expressed as median (25–75% quartiles) or numbers (%). *p* < 0.05 was considered statistically significant. * PCOS defined according to ESHRE 2003. ^a^ The period between the beginning of trying for a baby and confirmation of a pregnancy (assessed only in the women who gave birth). Abbreviations: A4, androstenedione; AMH, anti-Müllerian hormone; BMI, body mass index; DHEAS, dehydroepiandrosterone sulfate; FAI, free androgen index; FSH, follicle-stimulating hormone; HDL-c, high-density lipoprotein cholesterol; HOMA-IR, homeostasis model assessment for insulin resistance; HOMA-%β, homeostasis model assessment β-cell function; LH, luteinizing hormone; NS, statistically nonsignificant; PCOS, polycystic ovary syndrome; SHBG, sex hormone-binding globulin; TT, total testosterone; 17-OHP, 17-hydroxyprogesterone.

**Table 6:** Predictors of PCOS * persistence.

Covariates—Parameters Stated at the Beginning of the Follow-Up	OR	95% CI for OR		*p*-Value
		Lower	Upper	
Age at the follow-up ** (years)	0.84	0.72	0.98	0.03
BMI (kg/m^2^)	1.05	0.94	1.17	NS
Waist circumference (cm)	1.03	0.98	1.08	NS
Glucose 0′ (mg/dL)	1.03	0.94	1.14	NS
Insulin 0′ (uIU/mL)	1.09	0.98	1.21	NS
M-clamp value (mg/kgffm/min)	0.88	0.70	1.09	NS
Metabolic syndrome according to IDF	4.62	0.47	45.39	NS
hsCRP (mg/L)	1.07	0.87	1.31	NS
E-selectin (ng/mL)	1.08	1.01	1.17	0.03
Clinical hyperandrogenism	0.86	0.21	3.58	NS
LH (mIU/mL)	1.07	0.89	1.29	NS
FSH (mIU/mL)	0.39	0.20	0.78	0.007
TT (ng/mL)	0.56	0.05	6.57	NS
SHBG (nmol/L)	0.99	0.96	1.03	NS
FAI	1.06	0.88	1.28	NS
AMH (ng/mL)	1.19	0.99	1.43	0.06
Prolactin (ng/mL)	1.02	0.91	1.15	NS

Univariate logistic regression. Values are ORs (with 95% CI) and reflect the associations between the risk of PCOS persistence and investigated variables. *p* < 0.05 was considered statistically significant. * PCOS defined according to ESHRE 2018. ** The value of age assessed at the end of the follow-up was used in the analysis. Abbreviations: AMH, anti-Müllerian hormone; BMI, body mass index; CI, confidence interval; FAI, free androgen index; FSH, follicle-stimulating hormone; hsCRP, high-sensitivity C-reactive protein; IDF, International Diabetes Federation; LH, luteinizing hormone; M-clamp value, insulin sensitivity estimated with the euglycemic hyperinsulinemic clamp technique; NS, statistically nonsignificant; OR, odds ratio; PCOS, polycystic ovary syndrome; SHBG, sex hormone-binding globulin; TT, total testosterone.

## Data Availability

The data presented in this study are available on request from the corresponding author. The data are not publicly available due to the privacy of individuals that participated in the study.

## References

[1] Bozdag G., Mumusoglu S., Zengin D., Karabulut E., Yildiz B.O. (2016). The prevalence and phenotypic features of polycystic ovary syndrome: A systematic review and meta-analysis. Hum. Reprod..

[2] Lizneva D., Suturina L., Walker W., Brakta S., Gavrilova-Jordan L., Azziz R. (2016). Criteria, prevalence, and phenotypes of polycystic ovary syndrome. Fertil. Steril..

[3] Stein I.F., Leventhal M.L. (1935). Amenorrhea associated with bilateral polycystic ovaries. Am. J. Obstet. Gynecol..

[4] Conway G., Dewailly D., Diamanti-Kandarakis E., Escobar-Morreale H.F., Franks S., Gambineri A., Kelestimur F., Macut D., Micic D., Pasquali R. (2014). The polycystic ovary syndrome: A position statement from the European Society of Endocrinology. Eur. J. Endocrinol..

[5] Group Rotterdam ESHRE/ASRM-Sponsored PCOS Consensus Workshop (2004). Revised 2003 consensus on diagnostic criteria and long-term health risks related to polycystic ovary syndrome. Fertil. Steril..

[6] Teede H.J., Misso M.L., Costello M.F., Dokras A., Laven J., Moran L., Piltonen T., Norman R.J., Andersen M., Azziz R. (2018). Recommendations from the international evidence-based guideline for the assessment and management of polycystic ovary syndrome. Hum. Reprod..

[7] Azziz R., Carmina E., Dewailly D., Diamanti-Kandarakis E., Escobar-Morreale H.F., Futterweit W., Janssen O.E., Legro R.S., Norman R.J., Taylor A.E. (2006). Positions statement: Criteria for defining polycystic ovary syndrome as a predominantly hyperandrogenic syndrome: An Androgen Excess Society guideline. J. Clin. Endocrinol. Metab..

[8] Teede H.J., Deeks A., Moran L. (2010). Polycystic ovary syndrome: A complex condition with psychological, reproductive and metabolic manifestations that impacts on health across the lifespan. BMC Med..

[9] Plymate S.R., Matej L.A., Jones R.E., Friedl K.E. (1988). Inhibition of sex hormone-binding globulin production in the human hepatoma (Hep G2) cell line by insulin and prolactin. J. Clin. Endocrinol. Metab..

[10] Le T.N., Nestler J.E., Strauss J.F., Wickham E.P. (2012). Sex hormone-binding globulin and type 2 diabetes mellitus. Trends Endocrinol. Metab..

[11] Ding E.L., Song Y., Manson J.E., Hunter D.J., Lee C.C., Rifai N., Buring J.E., Gaziano J.M., Liu S. (2009). Sex hormone-binding globulin and risk of type 2 diabetes in women and men. N. Engl. J. Med..

[12] Burke C.W., Anderson D.C. (1972). Sex-hormone-binding globulin is an oestrogen amplifier. Nature.

[13] Poretsky L. (1991). On the paradox of insulin-induced hyperandrogenism in insulin-resistant states. Endocr. Rev..

[14] Diamanti-Kandarakis E., Dunaif A. (2012). Insulin resistance and the polycystic ovary syndrome revisited: An update on mechanisms and implications. Endocr. Rev..

[15] Rojas J., Chávez M., Olivar L., Rojas M., Morillo J., Mejías J., Calvo M., Bermúdez V. (2014). Polycystic ovary syndrome, insulin resistance, and obesity: Navigating the pathophysiologic labyrinth. Int. J. Reprod. Med..

[16] Rodriguez Paris V., Bertoldo M.J. (2019). The Mechanism of Androgen Actions in PCOS Etiology. Med. Sci..

[17] Livadas S., Pappas C., Karachalios A., Marinakis E., Tolia N., Drakou M., Kaldrymides P., Panidis D., Diamanti-Kandarakis E. (2014). Prevalence and impact of hyperandrogenemia in 1218 women with polycystic ovary syndrome. Endocrine.

[18] Keefe C.C., Goldman M.M., Zhang K., Clarke N., Reitz R.E., Welt C.K. (2014). Simultaneous measurement of thirteen steroid hormones in women with polycystic ovary syndrome and control women using liquid chromatography-tandem mass spectrometry. PLoS ONE..

[19] Pappalardo M.A., Vita R., Di Bari F., Le Donne M., Trimarchi F., Benvenga S. (2017). Gly972Arg of IRS-1 and Lys121Gln of PC-1 polymorphisms act in opposite way in polycystic ovary syndrome. J. Endocrinol. Invest..

[20] Yang R., Yang S., Li R., Liu P., Qiao J., Zhang Y. (2016). Effects of hyperandrogenism on metabolic abnormalities in patients with polycystic ovary syndrome: A meta-analysis. Reprod. Biol. Endocrinol..

[21] Patel S. (2018). Polycystic ovary syndrome (PCOS), an inflammatory, systemic, lifestyle endocrinopathy. J. Steroid Biochem. Mol. Biol..

[22] Anagnostis P., Tarlatzis B.C., Kauffman R.P. (2018). Polycystic ovarian syndrome (PCOS): Long-term metabolic consequences. Metabolism.

[23] Jacewicz-Święcka M., Kowalska I. (2018). Polycystic ovary syndrome and the risk of cardiometabolic complications in longitudinal studies. Diabetes Metab. Res. Rev..

[24] Fernández-Real J.M., Ricart W. (2003). Insulin resistance and chronic cardiovascular inflammatory syndrome. Endocr. Rev..

[25] Tarkun I., Arslan B.C., Cantürk Z., Türemen E., Sahin T., Duman C. (2004). Endothelial dysfunction in young women with polycystic ovary syndrome: Relationship with insulin resistance and low-grade chronic inflammation. J. Clin. Endocrinol. Metab..

[26] Kravariti M., Naka K.K., Kalantaridou S.N., Kazakos N., Katsouras C.S., Makrigiannakis A., Paraskevaidis E.A., Chrousos G.P., Tsatsoulis A., Michalis L.K. (2005). Predictors of endothelial dysfunction in young women with polycystic ovary syndrome. J. Clin. Endocrinol. Metab..

[27] Paradisi G., Steinberg H.O., Hempfling A., Cronin J., Hook G., Shepard M.K., Baron A.D. (2001). Polycystic ovary syndrome is associated with endothelial dysfunction. Circulation.

[28] Poredos P. (2002). Endothelial dysfunction in the pathogenesis of atherosclerosis. Int. Angiol..

[29] Kowalska I., Straczkowski M., Szelachowska M., Kinalska I., Prokop J., Bachórzewska-Gajewska H., Stepien A. (2002). Circulating E-selectin, vascular cell adhesion molecule-1, and intercellular adhesion molecule-1 in men with coronary artery disease assessed by angiography and disturbances of carbohydrate metabolism. Metabolism.

[30] Roldán V., Marín F., Lip G.Y., Blann A.D. (2003). Soluble E-selectin in cardiovascular disease and its risk factors. A review of the literature. Thromb. Haemost..

[31] Kado S., Nagata N. (1999). Circulating intercellular adhesion molecule-1, vascular cell adhesion molecule-1, and E-selectin in patients with type 2 diabetes mellitus. Diabetes Res. Clin. Pract..

[32] Diamanti-Kandarakis E., Paterakis T., Alexandraki K., Piperi C., Aessopos A., Katsikis I., Katsilambros N., Kreatsas G., Panidis D. (2006). Indices of low-grade chronic inflammation in polycystic ovary syndrome and the beneficial effect of metformin. Hum. Reprod..

[33] Victor V.M., Rovira-Llopis S., Bañuls C., Diaz-Morales N., de Marañon A.M., Rios-Navarro C., Alvarez A., Gomez M., Rocha M., Hernández-Mijares A. (2016). Insulin Resistance in PCOS Patients Enhances Oxidative Stress and Leukocyte Adhesion: Role of Myeloperoxidase. PLoS ONE.

[34] Victor V.M., Rocha M., Bañuls C., Alvarez A., de Pablo C., Sanchez-Serrano M., Gomez M., Rocha M., Hernández-Mijares A. (2011). Induction of oxidative stress and human leukocyte/endothelial cell interactions in polycystic ovary syndrome patients with insulin resistance. J. Clin. Endocrinol. Metab..

[35] Piouka A., Farmakiotis D., Katsikis I., Macut D., Gerou S., Panidis D. (2009). Anti-Mullerian hormone levels reflect severity of PCOS but are negatively influenced by obesity: Relationship with increased luteinizing hormone levels. Am. J. Physiol. Endocrinol. Metab..

[36] Diamanti-Kandarakis E. (2008). Polycystic ovarian syndrome: Pathophysiology, molecular aspects and clinical implications. Expert Rev. Mol. Med..

[37] Norman R.J., Dewailly D., Legro R.S., Hickey T.E. (2007). Polycystic ovary syndrome. Lancet.

[38] Cassar S., Teede H.J., Moran L.J., Joham A.E., Harrison C.L., Strauss B.J., Stepto N.K. (2014). Polycystic ovary syndrome and anti-Müllerian hormone: Role of insulin resistance, androgens, obesity and gonadotrophins. Clin. Endocrinol..

[39] Cook C.L., Siow Y., Brenner A.G., Fallat M.E. (2002). Relationship between serum müllerian-inhibiting substance and other reproductive hormones in untreated women with polycystic ovary syndrome and normal women. Fertil. Steril..

[40] Jonard S., Dewailly D. (2004). The follicular excess in polycystic ovaries, due to intra-ovarian hyperandrogenism, may be the main culprit for the follicular arrest. Hum. Reprod. Update.

[41] Casadei L., Fanisio F., Sorge R.P., Collamarini M., Piccolo E., Piccione E. (2018). The diagnosis of PCOS in young infertile women according to different diagnostic criteria: The role of serum anti-Müllerian hormone. Arch. Gynecol. Obstet..

[42] Zhao Y., Wang C., Liang Z., Liu X. (2019). Diagnostic Value of Anti-Müllerian Hormone as a Biomarker for Polycystic Ovary Syndrome: A Meta-Analysis Update. Endocr. Pract..

[43] Visser J.A., de Jong F.H., Laven J.S., Themmen A.P. (2006). Anti-Müllerian hormone: A new marker for ovarian function. Reproduction.

[44] Pigny P., Jonard S., Robert Y., Dewailly D. (2006). Serum anti-Mullerian hormone as a surrogate for antral follicle count for definition of the polycystic ovary syndrome. J. Clin. Endocrinol. Metab..

[45] Bhide P., Homburg R. (2016). Anti-Müllerian hormone and polycystic ovary syndrome. Best Pract. Res. Clin. Obstet. Gynaecol..

[46] Dewailly D., Andersen C.Y., Balen A., Broekmans F., Dilaver N., Fanchin R., Griesinger G., Kelsey T.W., La Marca A., Lambalk C. (2014). The physiology and clinical utility of anti-Mullerian hormone in women. Hum. Reprod. Update.

[47] Dumont A., Robin G., Catteau-Jonard S., Dewailly D. (2015). Role of Anti-Müllerian Hormone in pathophysiology, diagnosis and treatment of Polycystic Ovary Syndrome: A review. Reprod. Biol. Endocrinol..

[48] Zore T., Joshi N.V., Lizneva D., Azziz R. (2017). Polycystic Ovarian Syndrome: Long-Term Health Consequences. Semin. Reprod. Med..

[49] Balen A.H., Morley L.C., Misso M., Franks S., Legro R.S., Wijeyaratne C.N., Stener-Victorin E., Fauser B.C.J.M., Norman R.J., Teede H. (2016). The management of anovulatory infertility in women with polycystic ovary syndrome: An analysis of the evidence to support the development of global WHO guidance. Hum. Reprod. Update.

[50] Bahri Khomami M., Joham A.E., Boyle J.A., Piltonen T., Silagy M., Arora C., Misso M.L., Teede H.J., Moran L.J. (2019). Increased maternal pregnancy complications in polycystic ovary syndrome appear to be independent of obesity-A systematic review, meta-analysis, and meta-regression. Obes. Rev..

[51] Hudecova M., Holte J., Olovsson M., Sundström Poromaa I. (2009). Long-term follow-up of patients with polycystic ovary syndrome: Reproductive outcome and ovarian reserve. Hum. Reprod..

[52] Jacewicz-Święcka M., Kowalska I. (2020). Changes in Metabolic Profile in the Women with a History of PCOS-A Long-Term Follow-Up Study. J. Clin. Med..

[53] Kowalska I., Straczkowski M., Nikolajuk A., Adamska A., Karczewska-Kupczewska M., Otziomek E., Wołczyński S., Górska M. (2007). Serum visfatin in relation to insulin resistance and markers of hyperandrogenism in lean and obese women with polycystic ovary syndrome. Hum. Reprod..

[54] DeFronzo R.A., Tobin J.D., Andres R. (1979). Glucose clamp technique: A method for quantifying insulin secretion and resistance. Am. J. Physiol..

[55] Straczkowski M., Kowalska I., Nikolajuk A., Dzienis-Straczkowska S., Kinalska I., Baranowski M., Zendzian-Piotrowska M., Brzezinska Z., Górski J. (2004). Relationship between insulin sensitivity and sphingomyelin signaling pathway in human skeletal muscle. Diabetes.

[56] Swanson M., Sauerbrei E.E., Cooperberg P.L. (1981). Medical implications of ultrasonically detected polycystic ovaries. J. Clin. Ultrasound..

[57] Alberti K.G., Zimmet P., Shaw J., Group IETFC (2005). The metabolic syndrome--a new worldwide definition. Lancet.

[58] American Diabetes Association (2019). Classification and Diagnosis of Diabetes: Diabetes Care. Diabetes Care.

[59] Faddy M.J., Gosden R.G., Gougeon A., Richardson S.J., Nelson J.F. (1992). Accelerated disappearance of ovarian follicles in mid-life: Implications for forecasting menopause. Hum. Reprod..

[60] Tufan E., Elter K., Durmusoglu F. (2004). Assessment of reproductive ageing patterns by hormonal and ultrasonographic ovarian reserve tests. Hum. Reprod..

[61] Bili H., Laven J., Imani B., Eijkemans M.J., Fauser B.C. (2001). Age-related differences in features associated with polycystic ovary syndrome in normogonadotrophic oligo-amenorrhoeic infertile women of reproductive years. Eur. J. Endocrinol..

[62] Elting M.W., Korsen T.J., Rekers-Mombarg L.T., Schoemaker J. (2000). Women with polycystic ovary syndrome gain regular menstrual cycles when ageing. Hum. Reprod..

[63] Mulders A.G., Laven J.S., Eijkemans M.J., de Jong F.H., Themmen A.P., Fauser B.C. (2004). Changes in anti-Müllerian hormone serum concentrations over time suggest delayed ovarian ageing in normogonadotrophic anovulatory infertility. Hum. Reprod..

[64] Elting M.W., Kwee J., Korsen T.J., Rekers-Mombarg L.T., Schoemaker J. (2003). Aging women with polycystic ovary syndrome who achieve regular menstrual cycles have a smaller follicle cohort than those who continue to have irregular cycles. Fertil. Steril..

[65] Alsamarai S., Adams J.M., Murphy M.K., Post M.D., Hayden D.L., Hall J.E., Welt C.K. (2009). Criteria for polycystic ovarian morphology in polycystic ovary syndrome as a function of age. J. Clin. Endocrinol. Metab..

[66] Ahmad A.K., Kao C.N., Quinn M., Lenhart N., Rosen M., Cedars M.I., Huddleston H. (2018). Differential rate in decline in ovarian reserve markers in women with polycystic ovary syndrome compared with control subjects: Results of a longitudinal study. Fertil. Steril..

[67] Nikolaou D., Gilling-Smith C. (2004). Early ovarian ageing: Are women with polycystic ovaries protected?. Hum. Reprod..

[68] Macklon N.S., Fauser B.C. (2005). Ovarian reserve. Semin. Reprod. Med..

[69] Lockwood G.M., Muttukrishna S., Ledger W.L. (1998). Inhibins and activins in human ovulation, conception and pregnancy. Hum. Reprod. Update.

[70] Small C.M., Manatunga A.K., Klein M., Feigelson H.S., Dominguez C.E., McChesney R., Marcus M. (2006). Menstrual cycle characteristics: Associations with fertility and spontaneous abortion. Epidemiology.

[71] Brodin T., Bergh T., Berglund L., Hadziosmanovic N., Holte J. (2008). Menstrual cycle length is an age-independent marker of female fertility: Results from 6271 treatment cycles of in vitro fertilization. Fertil. Steril..

[72] Broekmans F.J., Soules M.R., Fauser B.C. (2009). Ovarian aging: Mechanisms and clinical consequences. Endocr. Rev..

[73] Dahlgren E., Johansson S., Lindstedt G., Knutsson F., Odén A., Janson P.O., Mattson L.A., Crona N., Lundberg P.A. (1992). Women with polycystic ovary syndrome wedge resected in 1956 to 1965: A long-term follow-up focusing on natural history and circulating hormones. Fertil. Steril..

[74] Orentreich N., Brind J.L., Rizer R.L., Vogelman J.H. (1984). Age changes and sex differences in serum dehydroepiandrosterone sulfate concentrations throughout adulthood. J. Clin. Endocrinol. Metab..

[75] Winters S.J., Talbott E., Guzick D.S., Zborowski J., McHugh K.P. (2000). Serum testosterone levels decrease in middle age in women with the polycystic ovary syndrome. Fertil. Steril..

[76] Morán C., Knochenhauer E., Boots L.R., Azziz R. (1999). Adrenal androgen excess in hyperandrogenism: Relation to age and body mass. Fertil. Steril..

[77] Birdsall M.A., Farquhar C.M. (1996). Polycystic ovaries in pre and post-menopausal women. Clin. Endocrinol..

[78] Pinola P., Piltonen T.T., Puurunen J., Vanky E., Sundström-Poromaa I., Stener-Victorin E., Ruokonen A., Puukka K., Tapanainen J.S., Morin-Papunen L.C. (2015). Androgen Profile Through Life in Women With Polycystic Ovary Syndrome: A Nordic Multicenter Collaboration Study. J. Clin. Endocrinol. Metab..

[79] Brown Z.A., Louwers Y.V., Fong S.L., Valkenburg O., Birnie E., de Jong F.H., Fauser B.C.J.M., Laven J.S.E. (2011). The phenotype of polycystic ovary syndrome ameliorates with aging. Fertil. Steril..

[80] Forslund M., Schmidt J., Brännström M., Landin-Wilhelmsen K., Dahlgren E. (2021). Reproductive Hormones and Anthropometry: A Follow-Up of PCOS and Controls From Perimenopause to Older Than 80 Years. J. Clin. Endocrinol. Metab..

[81] Hudecova M., Holte J., Moby L., Olovsson M., Stridsberg M., Larsson A., Berglund L., Berne C., Sundström-Poromaa I. (2011). Androgen levels, insulin sensitivity, and early insulin response in women with polycystic ovary syndrome: A long-term follow-up study. Fertil. Steril..

[82] Rittmaster R.S., Deshwal N., Lehman L. (1993). The role of adrenal hyperandrogenism, insulin resistance, and obesity in the pathogenesis of polycystic ovarian syndrome. J. Clin. Endocrinol. Metab..

[83] Pigny P., Merlen E., Robert Y., Cortet-Rudelli C., Decanter C., Jonard S., Dewailly D. (2003). Elevated serum level of anti-mullerian hormone in patients with polycystic ovary syndrome: Relationship to the ovarian follicle excess and to the follicular arrest. J. Clin. Endocrinol. Metab..

[84] Adams J., Polson D.W., Franks S. (1986). Prevalence of polycystic ovaries in women with anovulation and idiopathic hirsutism. Br. Med. J. (Clin. Res. Ed.).

[85] Carmina E., Campagna A.M., Lobo R.A. (2012). A 20-year follow-up of young women with polycystic ovary syndrome. Obstet Gynecol..

[86] Sundström I., Ildgruben A., Högberg U. (1997). Treatment-related and treatment-independent deliveries among infertile couples, a long-term follow-up. Acta Obstet. Gynecol. Scand..

[87] Koivunen R., Pouta A., Franks S., Martikainen H., Sovio U., Hartikainen A.L., McCarthy M.I., Ruokonen A., Bloigu A., Järvelin M.R. (2008). Fecundability and spontaneous abortions in women with self-reported oligo-amenorrhea and/or hirsutism: Northern Finland Birth Cohort 1966 Study. Hum. Reprod..

[88] West S., Vähäsarja M., Bloigu A., Pouta A., Franks S., Hartikainen A.L., Järvelin M.R., Corbett S., Vääräsmäki M., Morin-Papunen L. (2014). The impact of self-reported oligo-amenorrhea and hirsutism on fertility and lifetime reproductive success: Results from the Northern Finland Birth Cohort. Hum. Reprod..

[89] Szostak-Węgierek D., Waśkiewicz A., Piotrowski W., Stepaniak U., Pająk A., Kwaśniewska M., Nadrowski P., Niklas A., Puch-Walczak A., Drygas W. (2017). Metabolic syndrome and its components in Polish women of childbearing age: A nationwide study. BMC Public Health.

[90] Luo L., Gu F., Jie H., Ding C., Zhao Q., Wang Q., Zhou C. (2017). Early miscarriage rate in lean polycystic ovary syndrome women after euploid embryo transfer–A matched-pair study. Reprod. Biomed. Online.

[91] Mayrhofer D., Hager M., Walch K., Ghobrial S., Rogenhofer N., Marculescu R., Seemann R., Ott J. (2020). The Prevalence and Impact of Polycystic Ovary Syndrome in Recurrent Miscarriage: A Retrospective Cohort Study and Meta-Analysis. J. Clin. Med..

[92] Sagle M., Bishop K., Ridley N., Alexander F.M., Michel M., Bonney R.C., Beard R.W., Franks S. (1988). Recurrent early miscarriage and polycystic ovaries. BMJ.

[93] Rai R., Backos M., Rushworth F., Regan L. (2000). Polycystic ovaries and recurrent miscarriage--A reappraisal. Hum. Reprod..

[94] Rahman T.U., Ullah K., Guo M.X., Pan H.T., Liu J., Ren J., Jin L.Y., Zhou Y.Z., Cheng Y., Sheng J.Z. (2018). Androgen-induced alterations in endometrial proteins crucial in recurrent miscarriages. Oncotarget.

[95] Okon M.A., Laird S.M., Tuckerman E.M., Li T.C. (1998). Serum androgen levels in women who have recurrent miscarriages and their correlation with markers of endometrial function. Fertil. Steril..

[96] Pluchino N., Drakopoulos P., Wenger J.M., Petignat P., Streuli I., Genazzani A.R. (2014). Hormonal causes of recurrent pregnancy loss (RPL). Hormones.

[97] Ma L., Cao Y., Ma Y., Zhai J. (2021). Association between hyperandrogenism and adverse pregnancy outcomes in patients with different polycystic ovary syndrome phenotypes undergoing. Gynecol. Endocrinol..

[98] Tulppala M., Stenman U.H., Cacciatore B., Ylikorkala O. (1993). Polycystic ovaries and levels of gonadotrophins and androgens in recurrent miscarriage: Prospective study in 50 women. Br. J. Obstet. Gynaecol..

[99] Yang W., Yang R., Lin M., Yang Y., Song X., Zhang J., Yang S., Song Y., Li J., Pang T. (2018). Body mass index and basal androstenedione are independent risk factors for miscarriage in polycystic ovary syndrome. Reprod. Biol. Endocrinol..

[100] Bussen S., Sütterlin M., Steck T. (1999). Endocrine abnormalities during the follicular phase in women with recurrent spontaneous abortion. Hum. Reprod..

[101] Marques P., Ferreira F., Soares A.P., Nunes J., Sousa S., Aguiar A., Calhaz-Jorge C. (2016). Clinico-biochemical characteristics of 229 Portuguese infertile women with polycystic ovary syndrome: Clinical relevance and relationship with fertility treatment results. Clin. Exp. Obstet. Gynecol..

[102] Watson H., Kiddy D.S., Hamilton-Fairley D., Scanlon M.J., Barnard C., Collins W.P., Bonney R.C., Franks S. (1993). Hypersecretion of luteinizing hormone and ovarian steroids in women with recurrent early miscarriage. Hum. Reprod..

[103] Sun L., Lv H., Wei W., Zhang D., Guan Y. (2010). Angiotensin-converting enzyme D/I and plasminogen activator inhibitor-1 4G/5G gene polymorphisms are associated with increased risk of spontaneous abortions in polycystic ovarian syndrome. J. Endocrinol. Invest..

[104] Glueck C.J., Sieve L., Zhu B., Wang P. (2006). Plasminogen activator inhibitor activity, 4G5G polymorphism of the plasminogen activator inhibitor 1 gene, and first-trimester miscarriage in women with polycystic ovary syndrome. Metabolism.

[105] Bañuls C., Rovira-Llopis S., de Marañon A.M., Veses S., Jover A., Gomez M., Rocha M., Hernandez-Mijares A., Victor V.M. (2017). Metabolic syndrome enhances endoplasmic reticulum, oxidative stress and leukocyte-endothelium interactions in PCOS. Metabolism.

[106] Blumenfeld Z. (2019). The Possible Practical Implication of High CRP Levels in PCOS. Clin. Med. Insights Reprod. Health.

